# Experience with Oesophageal Cancer: A Ten-Year Single Centre Study Reflecting Daily Practice

**DOI:** 10.1155/2013/205417

**Published:** 2013-04-24

**Authors:** R. J. L. F. Loffeld, P. E. P. Dekkers

**Affiliations:** Department of Internal Medicine and Gastroenterology, Zaans Medical Centre, P.O. BOX 210, 1500 EE Zaandam, The Netherlands

## Abstract

*Introduction*. Studied patients with oesophageal cancer do not represent normal daily presentation. *Aim*. A retrospective study was done in all consecutive patients in order to describe presentation, treatment, and survival. *Patients*. All patients in a ten-year period were included. Patients were grouped in three groups. Group 1: no metastases and potentially curable, dead, or alive at time of evaluation. Group 2: patients presenting with metastases and treated with palliative chemotherapy, and group 3: patients with or without metastases but untreatable because of low Karnofsky index or important comorbidity rendering treatment not feasible. *Results*. One hundred thirty one evaluable patients were included. There was no difference in histological type of the tumour. Patients in group 3 were significantly older. Survival was not different between groups 2 and 3. Survival in group 1 was significantly longer (*P* < 0.0001) compared with groups 2 and 3. Patients in group 1 received treatment with chemoradiation and surgery. Patients in groups 2 and 3 were more often treated with palliative chemotherapy and endoscopic stenting. *Conclusion*. The overall survival of oesophageal cancer in normal daily life is poor. Supportive care seems to be the best treatment option in patients with metastases or low Karnofsky index. Palliative chemotherapy does not add to overall survival.

## 1. Introduction

Oesophageal cancer is diagnosed in about 400,000 patients each year worldwide, and its incidence is increasing. This makes it the ninth most common malignancy and sixth on the list of cancer mortality causes [1]. Studies on oesophageal cancer in the literature report on successful treatment: chemotherapy, radiotherapy, and surgery. Over the years the pattern of treatment options is changing. In the beginning of this century, surgery was the best option for reaching cure. The present standard therapy of potentially curable disease is the combination of neoadjuvant chemoradiation prior to surgical resection. Studies in the literature always include large numbers of treatable patients. However, this is not daily reality. In routine clinical daily practice, many patients present oesophageal cancer in a stage too advanced for curative therapy. Many patients do not show the initial presentation used as inclusion criterion in many clinical studies. Only a minority of the patients have potentially curable disease. It can be concluded that the studied population does not represent the normal patient population presenting with oesophageal cancer.

For this reason, a retrospective longitudinal study was done in all consecutive patients diagnosed with oesophageal cancer in a single centre in order to detect initial presentation and gain data on treatment and survival.

## 2. Patients and Methods

All consecutive patients undergoing upper gastrointestinal endoscopy in the Zaans Medical Centre, the community hospital of the Zaanstreek region in the Netherlands, in whom an oesophageal carcinoma was detected from 2001 until 2010, were included in the present study. Evaluation was done on December 31, 2011. Cases are always discussed in a multidisciplinary meeting with internists, oncologists, surgeons, gastroenterologists, and radiotherapists. In the first years of the study period, surgery was done in the Zaans Medical Centre by the local surgeons. Due to regionalisation of cancer treatment, surgery was done in a specific cancer clinic (Antoni van Leeuwenhoek ziekenhuis, Amsterdam, or the Academisch Medisch Centrum, Amsterdam) in the second half of the decennium. Neoadjuvant chemotherapy and palliative chemotherapy were done in the referral centre or by the local oncologist in the Zaans Medical Centre.

For all patients, files were searched for tumour type, initial stage of presentation, general condition of the patient, applied treatment, use of endoscopic stenting, and survival.

For the sake of the study, patients were grouped into three different groups. Group 1: patients presenting with treatable, localised, curable, and obviously nonmetastatic disease. This group was divided in two subgroups: group 1A, patients who died at time of the evaluation and group 1B, patients still alive at the moment of analysis at December 31, 2011. Group 2: patients with metastases at initial presentation and treated with palliative chemotherapy, and finally group 3: patients, with or without metastases, clinically judged as untreatable (unfit for curative treatment or palliative chemotherapy) by the multi-disciplinary team because of low Karnofsky index or important comorbidity.

Kaplan Meier curve survival analysis was used to compare survival times between groups 1, 2, and 3. Survival analyses were performed using the rms package, R version 2.13.1 (R foundation for statistical computing). Statistical analysis was done with chi-square test for contingency tables and *t*-test. A value below 0.05 was considered significant.

The study was approved by the Medical Ethics Committee of the Zaans Medical Centre.

## 3. Results

In the period of ten years, 138 patients were diagnosed with oesophageal cancer. From 6 patients (5 men, 1 women) no data (stage, general condition, and comorbidity) were available, because, after endoscopic diagnosis, they underwent further analysis and treatment elsewhere (4 adenocarcinomas, one squamous cell, and in one case no biopsies were taken because of use of anticoagulation therapy). From three of these patients, survival could be retrieved: 35, 417, and 109 days. One patient with an oesophageal cancer was diagnosed and treated elsewhere but visited the department because of food impaction in an oesophageal stent. Hence, 131 patients were evaluable.

A total of 48 patients (37%) presented with potentially curable disease. Sixty-four patients (49%) already had distant metastases at initial presentation, and 19 (15%) were unfit for any treatment.

In Table 1 the characteristics of the four groups of patients are shown. The majority of patients in all groups was male. There was no significant difference in histological type of the tumour in the four groups. Patients in group 3 were significantly older than patients in the other groups. However, from the range it will be clear that important overlap in age is present.

**Figure 1:**
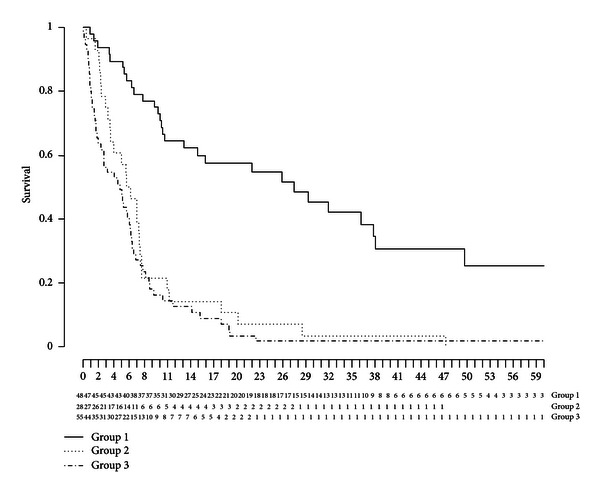
The survival in the three groups of patients. The *x*-axis shows the months. The *y*-axis shows the survival. Group 1 consists of censored data.

Table 2 shows the survival in days after the diagnosis in the four groups of patients. While in Table 3 the numbers surviving in years after the diagnosis are shown. In Figure 1 the Kaplan Meier curve with survival is shown from patients in groups 1, 2, and 3. Survival was not different between groups 2 and 3. Survival in group 1 was significantly longer (*P* < 0.0001) compared with groups 2 and 3. Survival in group 1B was significantly longer than in groups 1A, 2, and 3. From one patient in group 3 no survival data was present because he moved to another part of the country. As could be expected survival in group 1B was the longest; but on the other hand, not all these patients were treated and operated more than five years ago (censored data in the Kaplan Meier curve).

In group 1A three patients developed local recurrence after resection. Twelve patients developed metastases after their curative treatment. Ten patients died because of cardiac and pulmonary complications in the course of treatment, and from 8 patients the direct cause of death could not be determined anymore. The eighteen patients in group 1B were still alive at December 31, 2011. However, one of these patients developed metastases after treatment and is being treated with palliative chemotherapy. He lived 1437 days after the initial diagnosis. Two additional patients developed metastases during the course of preoperative chemotherapy and never underwent surgery. It can be expected that the life expectancy of these patients also will be limited.

Table 4 shows the endoscopic length of the tumour in the three groups of patients. In addition, the width of the tumour, this is the entire circumference or part of the circumference, is shown. There was no significant difference between the different groups. 

Table 5 shows the different treatment strategies applied in the patients. Obviously the patients in group 1 received the most aggressive treatment. Patients in groups 2 and 3 were significantly more often treated with palliative stenting. 

Table 6 shows the T and N stage in patients of group 1. There was no difference in T stage. However, N stage was significantly better in patients of group 1B (*P* < 0.001).  Table 7 shows the localisation of metastases in patients of groups 2 and 3.

Reasons for an expectative, palliative treatment in patients of group 3 were chronic obstructive lung disease 9 (*n* = 4), dementia (*n* = 1), severe cardiac disease (*n* = 4), advanced age (*n* = 12), bad general condition (*n* = 8), and decision to refute treatment (*n* = 2).

## 4. Discussion

The present population-based study reflects the experience with oesophageal cancer in normal routine daily clinical practice. All patients were treated according to the, at that moment, regionally accepted treatment modalities. 

All patients had oesophageal cancer. Obviously, this is always true for patients with squamous cell carcinoma. But, the gastric cardia was free of tumour in all patients especially in those with adenocarcinoma. The endoscopist was convinced of diagnosing oesophageal cancer and not in-growing stomach cancer.

In accordance with another Dutch study, the majority of patients had an adenocarcinoma [2]. The incidence of squamous cell carcinoma is decreasing in the past decennia, at least in the Western World [3]. 

From data in the literature, it is known that oesophageal cancer is lethal in most cases, yet its degree of aggressiveness varies from person to person [4]. This is also demonstrated in this study. Some patients, although these are exceptional cases, showed a long survival despite the fact that the disease was spread at presentation or the patient had a low Karnofsky index rendering treatment not feasible. Other patients died within a very short time after diagnosis (see Table 2).

The different treatment modalities applied in the patients of group 1 reflect the changing approach (from only surgery to the combination of chemoradiation and surgery) in the treatment of oesophageal cancer.

In the literature in 50–60% of the cases, patients have tumours that are considered inoperable, secondary to either tumour extension or medical co-morbidity [5]. The present data clearly show that this is even higher; 64% of patients are not curable or even untreatable at first presentation. The disease is advanced with distant lymph nodes or metastases, or the patient has co-morbidity rendering any curative option irrational. 

Figure 1 shows that there is no difference in survival whether the patient has metastases at diagnosis or is not treatable because of co-morbidity or low Karnofsky index. Almost all patients died within 21 months. The actual cause of death could not be determined, since many patients died at home. However, it is feasible to accept that death occurred as a direct consequence of the malignancy with disease progression in conjunction with the actual co-morbidity. It is tempting to suggest that the best treatment option in patients initially presenting with metastases of oesophageal cancer is best supportive care. Palliative chemotherapy or radiotherapy seems to have no direct advantage on survival in our patients. Survival is not better. However, there are no data on the effect of palliative therapy with respect to dysphagia or quality of life. On the other hand, it can be expected that palliative chemotherapy or radiotherapy will lead to potential toxicity impairing the quality of life. It could be argued that these treatment modalities should be applied cautiously in patients with metastases.

The five-year survival rate of oesophageal cancer after curative surgery improved to 40% according to statements in the literature [6]. Long-term survival in a Dutch study was only 20% [2]. These observations could not be confirmed in routine clinical practice. In fact, only 3.5% of patients in groups 1 and 3 were alive five years after diagnosis. In group 1B two patients were alive five years after the diagnosis. This figure can improve somewhat since 18 patients were still alive but only two reached the five-year survival. Probably the survival data in the literature are from highly selected patients (ASA1 or 2) or patients with very limited disease and very small tumours. Yoon et al. studied the influence of clinicopathological factors on survival in patients undergoing curative surgical resection [4]. They describe the importance of T status and tumour grade on survival. Patients with grades 2 and 3 showed the lowest survival despite the fact that the operation had curative intentions. T3 and T4 tumours had a five-year survival rate of around 20%. Tumour stage, this is, T1/T2 versus T3/T4 in groups 1A and 1B, was not different. But the N stage in group 1B was significantly lower. In daily practice endoscopic large tumours, this is over a longer segment of the oesophagus having mostly advanced stage cancers. This was not confirmed; there was no difference in endoscopic presentation between the different groups. Survival also is low in the presence of local regional lymph node involvement. In agreement with this study, patients above the age of 70 did worse [4].

A recent meta-analysis showed the superiority of neo-adjuvant chemoradiation and surgery versus surgery alone [7]. This meta-analysis reports on the odds ratios for survival but failed to show the actual survival rates in patients treated with resectable cancer. The overall survival improved, but numbers of patients alive more than 5 years after the treatment were not shown.

Only 37% of the patients in our hospital can be treated with curative intention after diagnosis. However, despite very aggressive therapy, the 5-year survival rate in patients of group 1, those who were potentially curable, is disappointingly low, only 2%. Of course this figure can be improved by including patients belonging to group 1B (still alive). Twenty-seven percent of patients who were potentially curable did not reach surgery because metastases became apparent during the course of neoadjuvant treatment. In younger patients survival is even worse [8]. 

One study reported on a five-year survival of 43% in patients with squamous cell carcinoma treated with 5-FU containing chemotherapy and radiotherapy [9]. This long survival could not be confirmed in the present study.

With the exception of some patients, it can be stated that the overall survival of oesophageal cancer in normal daily life is poor despite aggressive treatment in patients who are eligible. Supportive care seems to be the best treatment option in the majority of patients. Palliative chemotherapy does not add to overall survival. The opposite could be true; due to potential toxicity, quality of the remaining period of life could be decreased considerably. In addition, in times of financial crises and budget cuts, it can be argued that expensive therapy that does not add to survival or cure should not be used. 

The optimal treatment strategy for potentially curable oesophageal cancer still has to be established. It will be clear from this small study that despite differences in treatment strategies in the period of ten years, little change was seen in survival rates. 

## Figures and Tables

**Table 1:** Details of the four groups of patients.

	Number	m/f	A	S	Mean age	Median	Range
Group 1A	30	24/6	16	14	66	66	46–87
Group 1B	18	11/7	11	7	67	67	44–83
Group 2	28	24/4	19	9	64	64	37–87
Group 3	55	39/16	43	11	75	77	48–91

A: adenocarcinoma, S: squamous cell carcinoma, m: male, f: female, age in years.

**Table 2:** Survival in days after diagnosis in the four groups of patients.

Survival in days	Mean	SD	Median	Range
Group 1A	573	702	308	25–3540
Group 1B	1003	672	7632	350–2556
Group 2	261	299	179	11–1437
Group 3	199	288	145	1–1925

**Table 3:** Survival in years after the diagnosis.

	Start	1 year	2 years	3 years	4 years	5 years
Group 1A	30	12	9	5	2	1
Group 1B	18	17	9	6	4	2
Group 2	28	4	2	1	0	0
Group 3	55	7	7	1	1	1

**Table 4:** Endoscopic tumour length and distribution in the oesophagus.

	Group 1A	Group 1B	Group 2	Group 3
Endoscopic length				
<1 cm	12 (40%)	7 (39%)	(14%)	8 (15%)
1–3 cm	5 (17%)	4 (22%)	4 (14%)	8 (15%)
>3 cm	10 (33%)	7 (39%)	15 (54%)	31 (55%)
Unknown	3 (10%)	0	5 (18%)	8 (15%)
	*P* = ns			

Entire circumference	15 (50%)	8 (44%)	19 (68%)	36 (65%)
Half of the circumference	10 (33%)	6 (33%)	4 (14%)	11 (20%)
Unknown	5 (17%)	4 (23%)	5 (18%)	8 (15%)
	*P* = ns	

**Table 5:** Treatment strategies applied in the different groups.

	Surg	ch/RT/S	Chemo	RT	Supportive care	Stenting
Group 1A	17	4	2	5	0	3
Group 1B	9	5	1	1	0	1
Group 2	0	0	22	6	0	8
Group 3	0	0	0	0	55	32

Surg: surgery alone, ch/RT/S: neoadjuvant chemoradiation prior to surgery, chemo: chemotherapy alone, RT: radiotherapy alone.

One patient in group 1A was treated with endoscopic mucosal resection. One patient had preoperative chemotherapy alone. One patient in group 1B received a covered oesophageal expandable stent in order to seal an oesophageal perforation, prior to successful neoadjuvant therapy. Three patients in group 1A received endoscopic stenting because of local recurrence after successful initial treatment. Two patients in group 1B were treated with the combination of chemotherapy and radiotherapy.

Neoadjuvant chemoradiation consisted of radiation in a four-week schedule in combination with cisplatin 100 mg/m^2^ and 5-FU 1000 mg/m^2^.

**Table 6:** Tumour and lymph node stage.

	Tumour stage						
	T1	T2	T3	T4	N0	N1	Unknown
Group 1A	2	12	14	0	16	12	2
Group 1B	0	11	5	2	16	1	0

**Table 7:** Metastases in patients of groups 2 and 3.

	Group 2	Group 3
Mediastinal nodes	17	24
Supraclavicular nodes	4	4
Intra-abdominal nodes	12	12
Liver metastases	17	14
Lung metastases	1	6
Bone metastases	0	1
Peritonitis	1	0
Pleuritis	0	3
	*P* = ns	
